# Electrospinning Silk-Fibroin-Based Fibrous Membranes with AgNPs for Antimicrobial Application

**DOI:** 10.3390/polym16050648

**Published:** 2024-02-28

**Authors:** Qing Li, Hongyu Gong, Xiang Jia, Ran Wang, Zhiwei Liu, Lexin Zhang, Jisheng Li, Tifeng Jiao

**Affiliations:** 1 Hebei Key Laboratory of Safety Monitoring of Mining Equipment, School of Emergency Equipment, North China Institute of Science and Technology, Langfang 065201, China; 2 State Key Laboratory of Metastable Materials Science and Technology, Yanshan University, Qinhuangdao 066004, China; 3 Hebei Universities Characteristic Sericulture Application Technology Research and Development Center, Sericulture Research Institute, Chengde Medical University, Chengde 067000, China

**Keywords:** silk fibroin fibers, polyethylene oxide, AgNPs, antimicrobial application

## Abstract

Silk fibroin (SF) has excellent biocompatibility and is one of the most commonly used polymer materials. However, SF fibers have serious drawbacks as antibacterial materials due to their lack of stability and bacterial resistance. Therefore, it is of paramount significance to enhance the stability and bolster the bacterial resistance of SF fibers. In this study, SF fibers were fabricated and loaded with Ag nanoparticles (AgNPs) to improve the antimicrobial properties of the fibers. The impact of reduction conditions on the size of AgNPs was also investigated. In an antibacterial test, the fibers that were prepared exhibited over 98% bacterial resistance against *Staphylococcus aureus* (*S. aureus*) and *Escherichia coli* (*E. coli*). Therefore, as an efficient antibacterial material, these fibers are expected to become a candidate material in medical and textile fields. This study offers a novel approach for the utilization of SF fibers in the realm of antibacterial applications.

## 1. Introduction

In recent years, biomedical textiles have been rapidly developed [1], and they are extensively being applied in areas such as infection prevention [2], wound care [3], and biological tissue scaffolding [4]. Fibers prepared through electrospinning are considered to be ideal biomedical textiles owing to their large surface area [5], high porosity [6], and uniform fiber distribution [7]. However, their limitations in terms of electrical, thermal, and mechanical performance have hindered the development of fiber membranes via electrospinning. Mishra et al. utilized carbon nanotubes to reinforce PET fibers, ameliorating these deficiencies [8]. Furthermore, coaxial electrospinning, mixing and multiple electrospinning, core–shelled electrospinning, and blow-assisted electrospinning have expanded the possibilities of electrospinning technology in the field of medical textiles [9]. Electrospinning has emerged as a pivotal technique for fabricating nanoscale materials and devices, finding diverse applications in the realms of materials science, nanotechnology, biomedicine, textiles, and energy.

At present, fibers prepared from natural polymers retain the high biocompatibility advantages of these polymers [10]. Bazbouz et al. leveraged electrospinning to encapsulate ionic liquids and cellulose, preparing a dressing for the prevention of wound infection and promoting physiological healing [11]. Silk fibroin (SF), a natural protein, is widely used in medical dressings, tissue engineering, and other areas. Arumugam et al. synthesized silk fibroin/cellulose acetate/gold–silver nanoparticle (CA/SF/Au-Ag) fibers, which are considered one of the safer anticancer materials [12]. Foroushani et al. reported that silk fibroin–chitosan (CS)–Ag–curcumin (CUR) fibers could be used to enhance the pH-responsive release of CUR, accelerating wound care [13]. These studies collectively indicate that silk fibroin is a prime candidate as a natural polymer precursor for electrospinning. However, there are serious deficiencies when SF fibers are applied in the antimicrobial field. The brittleness [14] and antimicrobial properties [15] of silk fibers are both limiting factors in their development. The mechanical properties of artificially synthesized SF materials are inferior to those of other synthetic polymers. Furthermore, individual SF fibers exhibit relatively limited antimicrobial performance. It is imperative to overcome the shortcomings of SF fibers. 

The tear resistance of SF fibers could be improved via blending them with polyethylene oxide (PEO) [16]. The addition of PEO enhances the toughness and ductility of the fibers, thus imparting superior tensile performance. Li et al. prepared silk fibroin/polyethylene oxide (SF/PEO) scaffolds via electrospinning, which were combined with Sr-Cu-doped hollow bioactive glass nanospheres (Sr/Cu-HBGNs) loaded with vancomycin hydrochloride (VAN) [17]. The incorporation of PEO bolstered the tensile strength of this structure. This complex compound can extend the release time of pH-sensitive drugs. Lan et al. reported the influence of different SF and PEO ratios on SF/PEO fiber membranes [18]. An SF/PEO (3:7) composite demonstrated superior mechanical properties and exhibited remarkable efficacy in the loading and subsequent release of gentamicin sulfate (GS). This underscores the potential of GS/SF/PEO (3:7) as an outstanding antibacterial material. This innovative study delved into the impact of varying PEO proportions on the mechanical and antibacterial properties of the fibers, revealing that the addition of PEO does not follow a linear relationship regarding its benefits but rather exhibits an optimal value. Silver nanoparticles are considered highly efficient antibacterial and antiviral materials in biomedicine [19]. Algotiml et al. prepared different biogenic AgNPs with various extracts from seaweed and evaluated their physicochemical properties, cell activity, anticancer activities, and antibacterial activities [20]. The cited study revealed the potential of algal species extracts, especially *Ulva rigida* (*U. rigida*) extracts, which were effective reducing agents during the green synthesis of AgNPs. This implies that AgNPs can effectively integrate with natural materials and demonstrate excellent antibacterial properties. He et al. designed a carboxymethyl cellulose/cellulose nanocrystals@AgNPs (CMC/CNC@AgNPs)-coated paper, whose mechanical strength, water vapor and air barrier properties, and antibacterial activities were improved with the increase in the quantity of CNC@AgNPs [21]. AgNPs have made significant strides in the field of antibacterial resistance, including regarding their application in the electrospinning field. Fibers produced by electrospinning and decorated with nano-silver are widely used in textiles [22]. For example, Zhang et al. fabricated 12% Ag-doped ZnO fibers, which were shown to have excellent resistance to *E. coli* [23]. This work innovatively co-loaded ZnO and AgNPs onto the fiber membrane, endowing it not only with excellent antibacterial properties but also anti-inflammatory and bacteria-blocking effects. Mohammadi et al. applied AgNPs to hydrophobic polylactic acid (PLA) fibers after alkaline hydrolysis treatment [24]. Compared to regular PLA fibers, both AgNP-loaded PLA fibers (PLA-A) and AgNP-loaded hydrolyzed PLA fibers (PLA-H-A) exhibited highly porous structures, leading to a substantial enhancement in the antibacterial performance of the fibers. Magalhaes et al. also investigated the impact of AgNPs on the antibacterial performance of fiber membranes [25]. The results demonstrated that the synergistic action between AgNPs and the fiber membrane led to a reduction in bacteria of over 99.9%. These studies collectively demonstrate the excellent performance of the fiber membrane loaded with AgNPs in the field of antibacterial activity, providing a rich theoretical foundation and compelling practical evidence for enhancements of the antibacterial properties of SF fibers. Furthermore, the AgNPs reduced the permeation flux of the fiber membrane, mitigating the formation of cake layers on the membrane surface. However, compared to other antibacterial materials, further research is needed on fibers with AgNPs. The characteristics of PEO and AgNPs could overcome the drawbacks of SF, resulting in a high-strength, high-bacterial-resistance composite fiber membrane.

In the present study, we prepared SF/PEO fibers via electrospinning. By controlling the concentration of the reducing agent, that is, tannic acid (TA), and the reduction time, a series of SF/PEO/AgNPs fibers loaded with different amounts of AgNPs were obtained. The composition and morphology of the fibers were characterized, and their resistance to *S. aureus* and *E. coli* was studied. 

## 2. Materials and Methods

### 2.1. Materials and Processing

Silk fibroin, obtained from Chengde Medical University, was extracted from silkworm silk. Polyethylene oxide (PEO, M_w_ = 600,000 g mol^−1^), tannic acid (TA, M_w_ = 1701.2 g mol^−1^), formic acid (HCOOH, ≥99.5%), methanol (CH_3_OH, ≥99.7%), ethanol (EtOH, ≥99.7%), CaCl_2_ (≥96.0%), and AgNO_3_ (≥99.8%) were purchased from Aladdin reagents (Shanghai, China). Nutrient agar (NA) and nutrient broth (NB) were obtained from company Huankai Microbial (Guangzhou, China) and the Aobox factory (Beijing, China), respectively.

The electrospinning setup comprised a 10 mL plastic syringe connected to a volumetric pump and a power supply that could create a potential difference between the 20 G nozzle of the syringe (acting as the electrode) and the collector (serving as the ground). Deposition was performed at ambient temperature with a working distance of 15 cm, a spinning volume of 2 mL, a field strength of 18 kV, a flow rate of 0.8 mL/h, and a humidity level of 40 ± 5%, unless otherwise stated. An aluminum plate was employed as the collector.

### 2.2. Preparation of SF

A ternary mixture system of CaCl_2_/EtOH/H_2_O was prepared, with a molar ratio of 1:2:8. The degummed and dewaxed SF was then weighed and added to the ternary mixture system at a mass ratio of 1:5. SF was added batchwise to the ternary mixture system and heated in a water bath at 70 °C for 6 h until the fibers were completely dissolved. After cooling to room temperature, the solution was poured into a dialysis bag with a molecular weight cut-off of 3500 Dalton and dialyzed in deionized water for 72 h to remove CaCl_2_, anhydrous ethanol, and other small molecular impurities. Upon completion of dialysis, the remaining liquid in the dialysis bag was poured into a beaker and placed in a −4 °C refrigerator for 24 h. The frozen fibroin was then freeze-dried in a freeze-dryer for 48 h, resulting in the final sponge-like SF.

### 2.3. Preparation of Fibers

In total, 1.084 g of SF and 0.05 g of PEO were immersed in 8 mL of HCOOH and stirred at 35 °C for 6 h. The solution was allowed to rest at 25 °C for 24 h, and then the obtained spinning solution was subjected to electrospinning to form fibrous membrane. Afterwards, fibers produced through electrospinning were dried at 60 °C for 24 h to remove the remaining organic solvent and water from the fiber membrane. Finally, the SF/PEO was prepared.

In order to obtain a more ordered β-folded structure, the prepared SF/PEO was placed in methanol for 30 min. The processed SF/PEO was divided into four equal parts, labeled 1#, 2#, 3#, and 4#. Parts 1# and 2# were immersed in 5 mg mL^−1^ of TA solution and allowed to rest for 1 h and 2 h, respectively. Parts 3# and 4# were submerged in 10 mg mL^−1^ of TA solution and allowed to rest for 1 h and 2 h, respectively. The samples processed with TA solution were repeatedly washed with DI water and then reacted in a 20 mg mL^−1^ of AgNO_3_ solution. Finally, fibers with different treatment times and concentrations (SF/PEO/AgNPs-1, SF/PEO/AgNPs-2, SF/PEO/AgNPs-3, and SF/PEO/AgNPs-4) were obtained.

### 2.4. Characterization

The crystalline-phase information of the fiber films was ascertained via X-ray diffraction (XRD) using a D/max-2500/PC instrument (Nippon Rigaku Corporation, Tokyo, Japan). The scan rate utilized was 5° min^−1^, ranging from 5° to 60°. The chemical bonds and functional group types present in the fibers were characterized using Fourier transform infrared spectroscopy (FTIR) performed using a Nicolet iS10 (Thermo Scientific, Waltham, MA, USA). A thermogravimetry analysis (TG, STA8000, Perkin Elmer, Waltham, MA, USA) was carried out to analyze the thermal stability of silk fibroin fibers from 30 °C to 600 °C under N_2_ flow with a heating rate of 10 °C min^−1^. X-ray photoelectron spectroscopy (XPS) conducted utilizing an Escalab 250Xi instrument (Thermo Scientific, Waltham, MA, USA) was employed to analyze the chemical state and binding energy of the elements within the fiber membranes. The surface topography and energy-dispersive spectrometry data (EDS) of fibers were observed under a scanning electron microscope (SEM, S-4800II, JOEL, Tokyo, Japan), while platinum was loaded onto the surfaces of samples before measurement.

### 2.5. Antibacterial Activity

*Staphylococcus aureus* (*S. aureus*, ATCC^®^ 25923) and *Escherichia coli* (*E. coil*, ATCC^®^ 25922) were used as test bacterial strains to evaluate the antimicrobial properties of SF/PEO/AgNPs fibers. Bacterial suspension with an OD_600_ value ranging between 0.4 and 0.5 was utilized to assess antimicrobial activity.

The agar diffusion method outlined by Mittal et al. was employed to evaluate the antimicrobial efficacy of the fabricated films against *S. aureus* and *E. coli* [26]. Four SF/PEO/AgNPs fiber discs with a diameter of 1 cm were illuminated by UV light for 1 h. SF/PEO/AgNPs discs, sterilized by UV light, were put into two 10 mL sterilized NA plates, respectively. A 1 mL bacterial suspension was mixed with 150 mL of NA at 45 °C. Subsequently, 10 mL of the mixture was poured onto agar blocks containing SF/PEO/AgNPs fibers. The plates with SF/PEO/AgNPs fibers were cultivated at 37 °C for 24 h. While finishing the cultivation, the width of the inhibition zone could be measured. 

In addition to the width of the inhibition zone, the growth curve of antimicrobial activity is also one of the measurements used to assess fibrous films [27]. A 25 µL bacterial suspension was cultured on 1g of fiber samples for 15 min. The specimens were immersed in 40 mL of physiological saline and agitated for 5 min. A 1 mL aliquot of the mixture was subjected to sequential dilution to produce five distinct dilutions at 1-fold, 10-fold, 10^2^-fold, 10^3^-fold, and 10^4^-fold, yielding solutions of varying concentrations. Dilutions of 1 mL with varying concentrations were individually mixed with NA plates and subsequently incubated at 37 °C for 24 h. While finishing the cultivation, the colonies were meticulously observed and quantified. The antimicrobial rates of the fibers were determined using the following equation:

*X* = (*A* − *B*)/*A* × 100%

where *X* represents the antimicrobial rate, and *A* and *B* represent the bacterial counts after dilution and after dilution following treatment with fiber membranes, respectively. The growth curve of beneficial bacteria is also one of the criteria for evaluating the antibacterial performance of a fiber membrane. A total of 5 mg of different fibers was immersed into 1 mL of bacterial suspension (both *E. coil* and *S. aureus*) and 50 mL of NB and shaken with a speed of 160 rpm at 37 °C. To calculate and plot the antimicrobial growth curve, 200 µL of the mixture used to measure the value of OD_600_ was extracted every 2 h.

### 2.6. Statistical Analysis

All experiments were conducted in triplicate, and the mean values ± standard deviations (SD) were ascertained. Statistical distinctions were assessed using one-way analysis of variance (ANOVA) (*p* < 0.05).

## 3. Results and Discussion

### 3.1. Fibers Characterization

**Figure 1:**
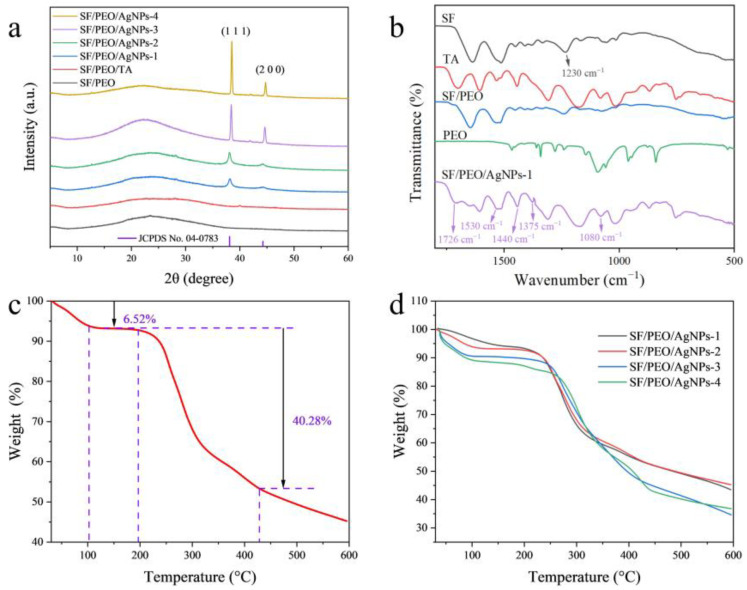
(**a**) XRD image of SF/PEO/AgNPs fibers; (**b**) FTIR image of SF/PEO/AgNPs-1 and other organic matter; TG images of (**c**) SF/PEO/AgNPs-1 and (**d**) SF/PEO/AgNPs fibers.

The XRD profiles of the four SF/PEO/AgNPs fibers are shown in Figure 1a. It can be seen that the SF/PEO/AgNPs fibers have diffuse peaks at a 2θ value of 24°, indicating the presence of the SF/PEO/TA substrate. The diffraction peaks corresponding to the Silk Ⅱ structure in SF are located at 28° and 36°, with their obscure appearances attributed to the disruption of the Silk Ⅱ structure by TA. The four fibers exhibit obvious diffraction peaks, centered at 39° and 45°, corresponding to the (200) and (100) planes of face-centered cubic structures from Ag [28], which proves that Ag^+^ was successfully reduced to Ag. Utilizing the Debye–Scherrer formula, it was determined that the particle sizes of the AgNPs loaded on the SF/PEOAgNPs-1~4 fibers were 0.584 nm, 0.611 nm, 0.363 nm, and 0.373 nm, respectively. The particle size of the AgNPs decreases with the increase in TA concentration, while the reaction time has minimal impact on the particle size. Under the influence of an increased concentration of reducing agent, the nucleation process of AgNPs may occur more rapidly, and the rate of particle growth is also accelerated, resulting in the formation of smaller particles during the reduction of Ag^+^. Furthermore, this phenomenon also indicates that the reduction of Ag^+^ was completed within one hour, leading to a consistent AgNP size as the reduction time increased.

In order to verify the interactions between molecules and the components within the membrane, the characteristic vibrative peaks in the main functional groups in SF/PEO/AgNPs-1 were confirmed using FTIR (Figure 1b). It is clear that the SF/PEO/AgNPs-1 displays characteristic peaks of TA, SF, PEO, and SF/PEO, which indicate that physical mixing would not disrupt the original properties of the polymers. The peak located at 1726 cm^−1^ was assigned to the C=O bond [29], indicating that changes occurred in the protein’s spatial conformation after the incorporation of TA into SF/PEO. The absorption peaks at 1630~1700 cm^−1^ and 1530 cm^−1^ are attributed to the bending vibrations of the C=O in the amide II band and the C−H in the amide I band of SF, respectively. The band at 1440 cm^−1^ corresponds to the stretching vibration of C−N. The band at 1375 cm^−1^ (C−O stretching) is significantly enhanced, which is attributed to the reaction of C=O and Ag^+^. During the reduction of Ag^+^ to AgNPs via TA, the C=O group in TA was oxidized to C−O, as evidenced by a distinct stretching vibration at 1375 cm^−1^ in the FTIR spectrum. The band at 1230 cm^−1^ primarily corresponds to the C−N stretching vibration and the N−H bending vibration of the amide III band in SF, while its absence in SF/PEO/AgNPs-1 may be attributed to the influence of thermal annealing. The band at 1080 cm^−1^ primarily represents the characteristic absorption peak of the C-O-C group in PEO. Furthermore, a comparative analysis of the FTIR spectra of TA with those of SF/PEO/AgNPs reveals a substantial presence of TA in the latter, indicating the complete reduction of Ag^+^ to AgNPs.

The TG results (Figure 1c,d) reveal the thermal stabilities of the SF/PEO/AgNPs fibers. As shown in Figure 1c, the weight of SF/PEO/AgNPs-1 reduces at temperatures of 100 °C, 200 °C, and 430 °C. Water and organic matter (approx. 6.52 wt.%) evaporate within the SF/PEO/AgNPs-1 from 30 °C to 100 °C. The temperatures of 200 °C and 430 °C correspond to the decomposition of proteins and the carbonization (approx. 40.28 wt.%) of fibrous films. The structural transition of amino acids between 100 °C and 200 °C signifies that there is no change in the quality of SF/PEO/AgNPs-1 within this temperature range. This arises from the disruption of the molecular structure of amino acids and the cross-linking of SF and TA. The SF/PEO/AgNPs-2~4 also exhibited a similar trend. At temperatures exceeding 600 °C, a substantial 45 wt.% residue persisted, an observation suggestive of the adherence of AgNPs to the fiber surfaces and the residual carbonization of fibers. However, with the increase in TA concentration, the initial degradation temperature of the fibers also increased (Figure 1d). This phenomenon indicates that the crosslinking of TA with SF could enhance the stability of the fibers. SF/PEO/AgNPs-3 and SF/PEO/AgNPs-4 each achieved the decomposition of proteins and the carbonization of fibrous membranes at 450 °C owing to the augmented presence of TA, thus enhancing the stability of the fibers. Furthermore, at 600 °C, SF/PEO/AgNPs-3 and SF/PEO/AgNPs-4 exhibited a reduced mass, attributed to the increased crosslinking of fibers facilitated by the greater presence of TA. The TG results indicate that the percentage of AgNPs in SF/PEO/AgNPs-1 and 2 were approx. 45%, while in SF/PEO/AgNPs-3 and 4, the percentage of AgNPs was approx. 35%. In conjunction with the XRD results, this suggests that although an increase in TA concentration can reduce the particle size of AgNPs, it also decreases the loading capacity of AgNPs. Simultaneously, the reduction time scarcely impacts the loading capacity of AgNPs.

**Figure 2:**
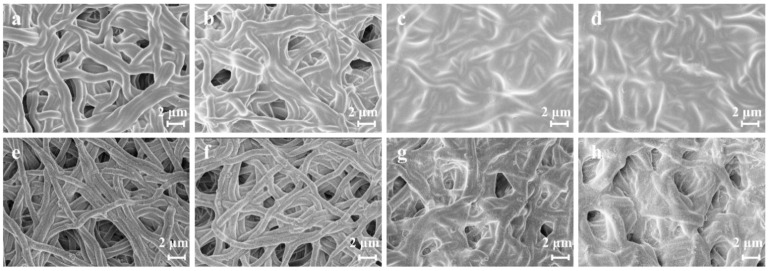
SEM images of SF/PEO fibers with (**a**) 1# ([TA] = 5 mg mL^−1^, time = 1 h), (**b**) 2# ([TA] = 5 mg mL^−1^, time = 2 h), (**c**) 3# ([TA] = 10 mg mL^−1^, time = 1 h), and (**d**) 4# ([TA] = 10 mg mL^−1^, time = 2 h). SEM images of (**e**) SF/PEO/AgNPs-1, (**f**) SF/PEO/AgNPs-2, (**g**) SF/PEO/AgNPs-3, and (**h**) SF/PEO/AgNPs-4.

**Figure 3:**
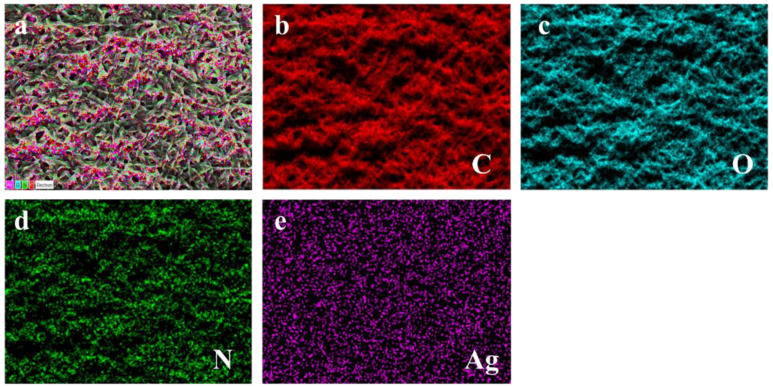
(**a**) EDS image of SF/PEO/AgNPs-1. Elemental mapping images of (**b**) C, (**c**) O, (**d**) N, and (**e**) Ag.

SEM information is shown in Figure 2. It can be observed in Figure 2a,b that, as the reaction time increases, the diameter of the fibers enlarges. This trend is also reflected in SF/PEO/AgNPs-3~4 (Figure 2c,d). However, with the increase in TA concentration and reaction time, SF undergoes crosslinking with TA, which is structurally manifested by a gradual blurring of the fiber structure and the complete disappearance of the pore structure. The introduction of Ag^+^ results in the re-emergence of a distinct microstructure and an evident pore structure in SF/PEO/AgNPs (Figure 2e–h). This is attributed to the reduction of Ag^+^ to AgNPs via the phenolic hydroxyl groups of TA, leading to a weakened interaction between TA and SF, which results in the extensive shedding of TA from SF/PEO/AgNPs. In order to visually ascertain the loading of AgNPs on the surface of SF/PEO/AgNPs-1, an EDS analysis was employed to determine their elemental distribution on its surface. The EDS images (Figure 3) substantiated the uniform loading of AgNPs on the surface of SF/PEO/AgNPs-1, aligning with the SEM and XRD findings.

**Figure 4:**
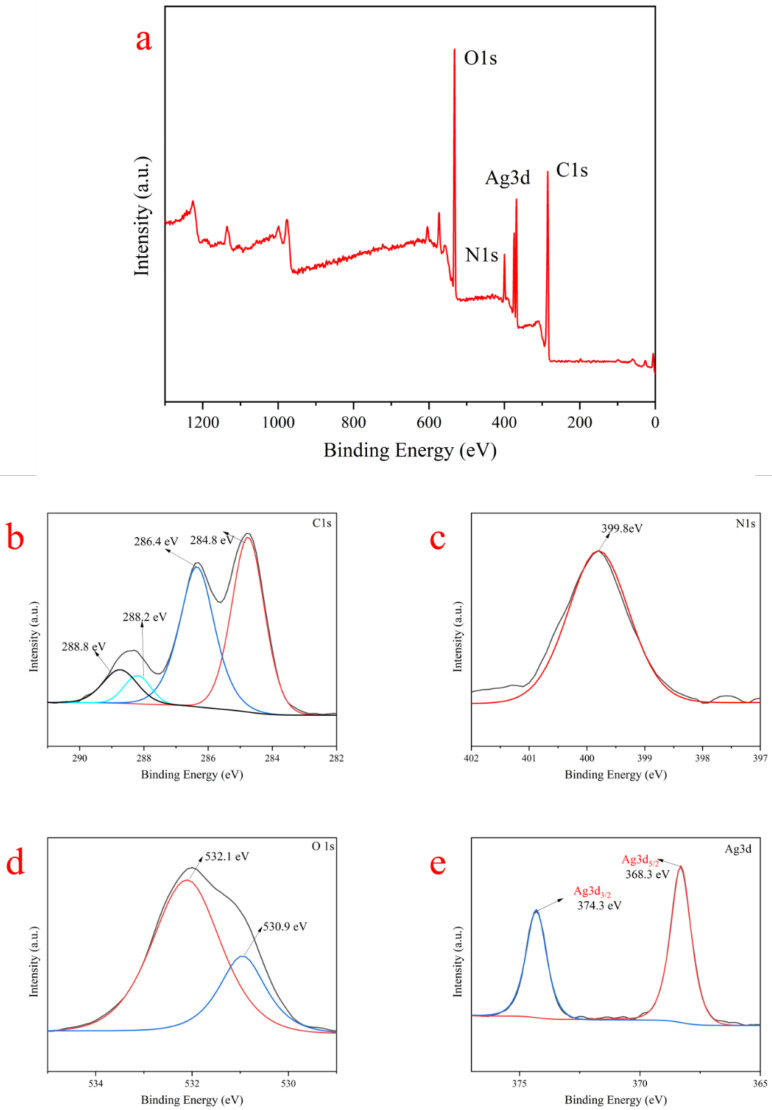
(**a**) XPS spectra of SF/PEO/AgNPs-1. High-resolution SF/PEO/AgNPs-1 XPS spectra of (**b**) C 1s, (**c**) N 1s, (**d**) O 1s, and (**e**) Ag 3d.

To further analyze the chemical valence state and surface composition of SF/PEO/AgNPs-1, an XPS analysis was carried out. Figure 4a shows the existence of C, O, N, and Ag elements in SF/PEO/AgNPs-1. In Figure 4b, the fitting curve of the high-resolution C 1s XPS spectra of SF/PEO/AgNPs-1 shows four C 1s peaks, which were ascribed to O−C=O (288.8 eV), C−N and C=C of benzene rings (288.2 eV), C−OH (286.4 eV), and alkane carbon (284.8 eV). Similarly, two O 1s peak signals were detected at 532.1 and 530.9 eV (Figure 4c), corresponding to 288.8 and 286.4 eV of the C 1s spectrum in Figure 4b. Furthermore, the peak at 399.8 eV of the N 1s spectra in Figure 4d was attributed to C−NH_2_ (acetamide), implying that more electronegative substituents exist on the nitrogen atom, including O−N and Ag−N. The Ag 3d spectrum of SF/PEO/AgNPs-1 displays two major peaks of Ag 3d_3/2_ (Ag^0^) and Ag 3d_5/2_ (Ag^0^) at 374.3 and 368.3 eV (Figure 4e), respectively [30]. This suggests that Ag is attached to the surface of SF/PEO in its elemental form.

### 3.2. Antibacterial Test

**Figure 5:**
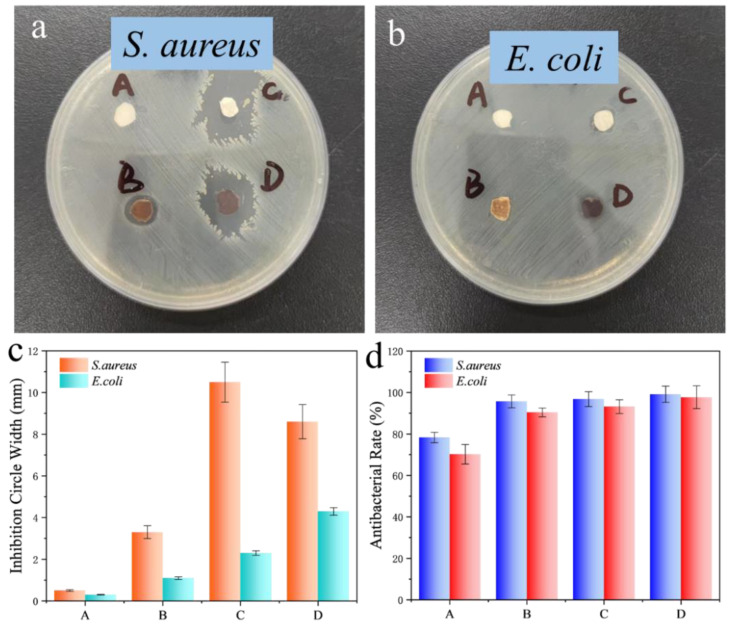
Zones of SF/PEO/AgNPs fibers against (**a**) *S. aureus* and (**b**) *E. coli*. Antimicrobial rate of SF/PEO/AgNPs fibers; (**c**) The width of the inhibition zone of four fibers; (**d**) The antibacterial rate of four fibers. (A: SF/PEO/AgNPs-1, B: SF/PEO/AgNPs-2, C: SF/PEO/AgNPs-3, and D: SF/PEO/AgNPs-4).

Figure 5a,b show the inhibition zones of SF/PEO/AgNPs fibers against different bacteria (*E. coli* and *S. aureus*). In these images, it can be seen that there are noticeable inhibition zones and a distinct brownish-yellow color, caused by the diffusion of TA and AgNPs. In addition, the width of the inhibition zone gradually increases with the increase in TA concentration and processing time (Figure 5c). This indicates that changes in TA treatment conditions will cause changes in the loading of AgNPs on SF/PEO/AgNPs fibers. The results regarding the inhibition zone width demonstrate that SF/PEO/AgNPs exhibit a stronger inhibitory effect on Staphylococcus aureus compared to Escherichia coli, a finding that was further corroborated in subsequent experiments. Four types of SF/PEO/AgNPs exhibit excellent resistance to *S. aureus* and *E. coli*, especially SF/PEO/AgNPs-4, with antimicrobial rates against both bacteria reaching higher than 98%, which exceeds the 78.3% and 70.2% of SF/PEO/AgNPs-1 (Figure 5d). Fibers from SF/PEO/AgNPs-1 to SF/PEO/AgNPs-4 show increasing resistance to *S. aureus* and *E. coli* because of the increasing content of Ag. Additionally, all the SF/PEO/AgNPs films display a better resistance to *S. aureus* than to *E. coli*. This enhanced resistance is attributed to the complex cell wall structure of Gram-negative bacteria, which could form a barrier preventing antibiotics from entering.

**Figure 6:**
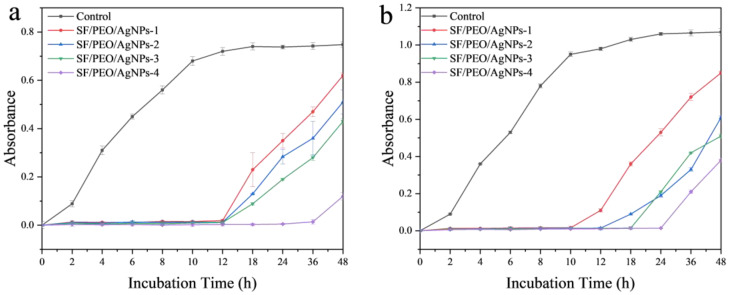
Growth curves of (**a**) *S. aureus* and (**b**) *E. coli*.

The antimicrobial growth curve of SF/PEO/AgNPs fibers against *S. aureus* is shown in Figure 6a. Benefiting from a higher loading of AgNPs, SF/PEO/AgNPs-4 exhibits a better inhibitory effect on *S. aureus* (36 h) compared with that of SF/PEO/AgNPs-1 to SF/PEO/AgNPs-3 (12 h). This is due to the significantly higher loading of AgNPs on the SF/PEO/AgNPs-4 fiber membrane compared to that of the other three membranes, resulting in superior antibacterial efficacy of SF/PEO/AgNPs-4. However, the inhibitory effect of SF/PEO/AgNPs fibers on *E. coli* is weaker than that on *S. aureus* (Figure 6b), as shown in the results of the inhibition zone experiment (Figure 5a,b). Similar to the experimental results for *S. aureus*, SF/PEO/AgNPs-4 has the best inhibitory effect on *E. coli* (24 h). Moreover, the greater the loading of AgNPs, the higher the SF/PEO/AgNPs fibers’ resistance to *E. coli*. After 12 h, the growth of *E. coli* commenced, which could be attributed to the detachment of silver nanoparticles loaded on the composite nanofibers within 0–12 h (Figure 6b), which exerted an inhibitory effect on the growth of Escherichia coli. However, over time, the release of AgNPs gradually slowed, leading to a diminished inhibitory effect on the bacteria. In comparison with similar types of Ag-loaded fibers [31,32,33], the SF/PEO/AgNPs exhibit superior antimicrobial performance while retaining the characteristics of fibroin, thus offering a broader range of potential applications. One of the key reasons for the widespread application of SF in the medical textile field is its excellent biocompatibility [34]. Leveraging this characteristic, numerous outstanding fibrous membranes have been designed for use in the biomedical field [35,36,37]. However, the potential suitability of the SF/PEO/AgNPs fibrous membrane that we prepared for this field remains to be evaluated, warranting further experimental validation. This presents a new avenue for our future research.

## 4. Conclusions

In this work, a sample method for the in situ reduction of silver with the active polyphenol TA was designed to fabricate the SF/PEO/AgNPs fibers via electrospinning. The obtained membranes were characterized analytically. The XRD results indicated that the particle sizes of AgNPs in SF/PEO/AgNPs-1~4 were 0.584 nm, 0.611 nm, 0.363 nm, and 0.373 nm, respectively. The TG revealed the percentage of AgNPs in SF/PEO/AgNPs-1~2 (approx. 45 wt.%) and SF/PEO/AgNPs-3~4 (approx. 35 wt.%). The SEM images not only displayed the microstructure of SF/PEO/AgNPs but also elucidated the influence of the fiber structure with different TA concentrations and treatment times. The XPS data confirmed the chemical state of Ag on the surfaces of the fibers. The antibacterial tests demonstrated that SF/PEO/AgNPs-4, with higher AgNP content, exhibited stronger inhibition against *S. aureus* (99.2%) and *E. coli* (98.0%). The SF/PEO/AgNPs fibers’ longer inhibition time was ascribed to the simple cell wall structure of *S. aureus*. This study provides a new perspective for research on SF fibers as a potential antibacterial material.

## Data Availability

The raw data supporting the conclusions of this article will be made available by the authors on request.
